# Supplementary material for: Impact of COVID-19 vaccination on liver transplant recipients. Experience in a reference center in Mexico
Source: PLoS One. 2024 Mar 28;19(3):e0301198. doi: 10.1371/journal.pone.0301198 (PMC10977796; doi:10.1371/journal.pone.0301198)
Supplement: Attachment — Submitted filename: Response to Reviewers.docx [file pone.0301198.s001.docx]

Mexico City, Mexico, January 11, 2024

Dear Reviewers and editor,

First, we thank you for your points of view and the remarks that you made regarding our study. Here, we respond to your requests and observations.

**Reviewer #1:**

1. I was very happy because the topic presented in the article was from an area that I was specifically interested in.
   Authors ''The risk in liver transplant (LT) patients
   "has not been widely investigated," they said. This statement is unrealistic in my opinion. There are very serious studies written on this subject.
   a. **The paragraph was changed to acknowledge research that was recently published.**
      i. **Background and aims**: COVID-19 vaccination has proven to be effective in preventing symptomatic infection and severe disease, even in immunocompromised patients, including liver transplant patients. We aimed to assess the impact of the COVID-19 vaccination on mortality and the development of severe and critical disease in our center.
2. Please use the following studies to enrich the introduction and discussion sections of your article:

   Akbulut S, Yagin FH, Sahin TT, Garzali IU, Tuncer A, Akyuz M, Bagci N, Barut B, Unsal S, Sarici KB, Saritas S, Ozer A, Bentli R, Colak C, Bayindir Y, Yilmaz S. Effect of COVID-19 Pandemic on Patients Who Have Undergone Liver Transplantation: Retrospective Cohort Study. J Clin Med. 2023;12(13):4466.
   a. **This article has been cited in the Discussion section (reference number 25).**
      i. This was also found in a study from Turkey with 387 LT recipients with confirmed COVID, and tacrolimus was found to be a protective factor against death due to COVID 19 (HR 3.44 (95% CI = 1.35–8.33)). They also found that immunosuppression with everolimus (HR 2.94 (95% CI = 1.35–6.42)) and prednisolone (2.53 fold (95% CI = 1.01–6.06)) was associated with a higher mortality; they found no effect on MMF [24].

Akbulut S, Bagci N, Akyuz M, Garzali IU, Saritas H, Tamer M, Ince V, Unsal S, Aloun A, Yilmaz S. Effect of COVID-19 Pandemic on Patients Who Have Undergone Liver Transplantation Because of Hepatocellular Carcinoma. TransplantProc. 2023;55(5):1226-1230.

a. **This article has been cited in the Introduction section (reference number 8).**
   i. Liver transplant (LT) patients do not appear to be more prone to COVID-19 infection, with a similar incidence rate as the general population (3.18 cases/100 person-years in LT patients vs. 3.97 cases/100 person-years in non-LT patients), also featuring lower in-hospital mortality (18% vs. 27%) [5–8].

In response to the reviewer's feedback, we thoroughly examined the other references suggested for our paper. However, upon careful analysis, we concluded that these references were not pertinent or directly relevant to the content and focus of our paper. It's crucial to note that the decision was based on a comprehensive evaluation of the suggested sources in relation to the specific objectives, scope, and context of our research.

3. Delete the "Overall" column in the tables. Leave only the columns belonging to the two groups and the column where the "p" value is given in the table.
   a. **The overall column has been deleted from all the tables.**
4. In the latest study published by Akbulut et al. (PMID: 37445501; J Clin Med. 2023 Jul 3;12(13):4466.), the factors affecting mortality in atransplant patients were seriously examined and it was stated that vaccination reduced mortality by 100 times. In the same study, immunosuppressive drugs and comorbidities were shown to be associated with mortality. It is very important to comment on this study and other large studies like it in the discussion section.
   a. **The following paragraph has been added to present the information demonstrated in this study.**
      i. In a retrospective study in Turkey, COVID-19 vaccination was associated with a 100-fold reduction in mortality in LT recipients; in this study, comorbidities such as diabetes mellitus and hypertension were not associated with higher mortality [25].
      ii. This was also found in a study from Turkey with 387 LT recipients with confirmed COVID, and tacrolimus was found to be a protective factor against death due to COVID 19 (HR 3.44 (95% CI = 1.35–8.33)). They also found that immunosuppression with everolimus (HR 2.94 (95% CI = 1.35–6.42)) and prednisolone (2.53 fold (95% CI = 1.01–6.06)) was associated with a higher mortality; they found no effect on MMF [25].
5. Please add a paragraph about the limitations of the study to the last part of the discussion.
   a. **We acknowledge our limitations and have added a subtitle to this part of the discussion.**
      i. We performed a single center study with the limitations that it carries like a limited number of patients. We do not know the COVID variant that these patients were infected with or their antibody titers. We attempted to control for potential confounders; however, because of the low mortality rate, we adjusted the regression models for only two variables.

**Reviewer #2:**

1. The authors assessed the impact of COVID-19 vaccination on the mortality and development of severe and critical disease in LT recipients. They found that vaccination reduces the risk of severe COVID-19 in LT patients, regardless of the scheme used. I think this conclusion is well acknowledged. So this topic seems lack of novelty to the readers of Plos One Journal.
   a. **We appreciate the input by the reviewer; however, we think that this study is of relevance because the study shows quality data of a high-volume reference center of liver transplant in a previously.**

**Journal Requirements:**

1. Please ensure that your manuscript meets PLOS ONE's style requirements, including those for file naming. The PLOS ONE style templates can be found at
   https://journals.plos.org/plosone/s/file?id=wjVg/PLOSOne_formatting_sample_main_body.pdf and 
   <https://journals.plos.org/plosone/s/file?id=ba62/PLOSOne_formatting_sample_title_authors_affiliations.pdf>
   a. **The style requirements have been met and the changes have been highlighted in the manuscript.**
2. Please provide additional details regarding participant consent. In the ethics statement in the Methods and online submission information, please ensure that you have specified (1) whether consent was informed and (2) what type you obtained (for instance, written or verbal, and if verbal, how it was documented and witnessed). If your study included minors, state whether you obtained consent from parents or guardians. If the need for consent was waived by the ethics committee, please include this information.
1. If you are reporting a retrospective study of medical records or archived samples, please ensure that you have discussed whether all data were fully anonymized before you accessed them and/or whether the IRB or ethics committee waived the requirement for informed consent. If patients provided informed written consent to have data from their medical records used in research, please include this information.

In this instance it seems there may be acceptable restrictions in place that prevent the public sharing of your minimal data. However, in line with our goal of ensuring long-term data availability to all interested researchers, PLOS’ Data Policy states that authors cannot be the sole named individuals responsible for ensuring data access ([http://journals.plos.org/plosone/s/data-availability#loc-acceptable-data-sharing-methods](http://journals.plos.org/plosone/s/data-availability#loc-acceptable-data-sharing-methods)).

a. **This information has been clarified in the methods section of our journal and a subtitle has been added to the ethics statement.**
   i. **2.1 Ethics statement**
      1. This study was conducted in accordance with the principles of the Declaration of Helsinki (World Medical Association Declaration of Helsinki Ethical Principles for Medical Research Involving Human Subjects, Version Fortaleza, Brazil, October 2013). This research was conducted in full compliance with the ethical guidelines and regulations set forth by the institutional research and ethics committee (*Comité de Investigación del Instituto Nacional de Ciencias Médicas y Nutrición Salvaodr Zubiran* and *Comité de Ética en Investigación del Insituto Nacional de Ciencias Médicas y Nutrición Salvador Zubiran*, registration number 09-CEI-011-20160627, approval number: REF. 3678; date of approval April 12^th^ 2021). As the information was analyzed anonymously and data were extracted from electronic medical records, consent was waived by the institutional research and ethics committee.

2. Data requests to a non-author institutional point of contact, such as a data access or ethics committee, helps guarantee long term stability and availability of data. Providing interested researchers with a durable point of contact ensures data will be accessible even if an author changes email addresses, institutions, or becomes unavailable to answer requests.

Before we proceed with your manuscript, please also provide non-author contact information (phone/email/hyperlink) for a data access committee, ethics committee, or other institutional body to which data requests may be sent. If no institutional body is available to respond to requests for your minimal data, please consider if there any institutional representatives who did not collaborate in the study, and are not listed as authors on the manuscript, who would be able to hold the data and respond to external requests for data access? If so, please provide their contact information (i.e., email address). Please also provide details on how you will ensure persistent or long-term data storage and availability.

a. **Contact information to the ethics committee to maintain long-lasting contact in response to external requests for data access.**
   i. **The Ethics committee of the Instituto Nacional de Ciencias Médicas y Nutricion Salvador Zubiran can be contacted through the email [comite.etica.investigacion@incmnsz.mx](mailto:comite.etica.investigacion@incmnsz.mx).**
b. **We changed the a data availability statement**
   i. All relevant data are within the manuscript.

3. Please include your full ethics statement in the ‘Methods’ section of your manuscript file. In your statement, please include the full name of the IRB or ethics committee who approved or waived your study, as well as whether or not you obtained informed written or verbal consent. If consent was waived for your study, please include this information in your statement as well.
   a. **We added the complete names of the committees that approved the study to the original language and reference number.**
      i. This research was conducted in full compliance with the ethical guidelines and regulations set forth by the institutional research and ethics committee (*Comité de Investigación del Instituto Nacional de Ciencias Médicas y Nutrición Salvaodr Zubiran* and *Comité de Ética en Investigación del Insituto Nacional de Ciencias Médicas y Nutrición Salvador Zubiran*, REF. 3678). As the information was analyzed anonymously and data were extracted from electronic medical records, consent was waived by the institutional research and ethics committee. This study adhered to the ethical principles outlined in the Declaration of Helsinki.
